# Informing retention in longitudinal cohort studies through a social marketing lens: Raine Study Generation 2 participants’ perspectives on benefits and barriers to participation

**DOI:** 10.1186/s12874-020-01074-z

**Published:** 2020-07-29

**Authors:** Leesa Costello, Julie Dare, Manon Dontje, Leon Straker

**Affiliations:** 1 School of Medical and Health Sciences, Edith Cowan University, Perth, WA Australia; 2 Department of Public Health, Erasmus MC, University Medical Center Rotterdam, Rotterdam, The Netherlands; 3 School of Physiotherapy and Exercise Science, Curtin University, Perth, WA Australia

**Keywords:** The Raine study, Longitudinal cohorts, Retention, Attrition, Participant experience, Social marketing, Qualitative interviews

## Abstract

**Background:**

Longitudinal cohort studies have made significant contributions to medical discoveries and provide the impetus for health interventions which reduce the risk of disease. Establishing and maintaining these cohorts is challenging and costly. While some attrition is unavoidable, maintaining a sufficient number of participants ensures that results remain representative and free from bias. Numerous studies have investigated ways to reduce attrition but few studies have sought to understand the experience of participants, and none have examined this through a social marketing framework. This first paper in a two part-series describes participants’ experiences according to: benefits, barriers, motivators and influencers. The second paper uses this understanding to address issues relating to the 4Ps (product, price, place, promotion) of social marketing.

**Methods:**

Participants were recruited from the Raine Study, a pregnancy cohort study that has been running in Western Australia since 1989. Qualitative interviews were conducted with 29 active and inactive participants from the Generation 2 cohort, who were originally enrolled in the Raine Study at birth by their parents (Generation 1). ‘Active’ participants (*n* = 17) were defined as those who agreed to attend their 27 year follow-up, while ‘inactive’ (*n* = 12) participants were defined as those who had not attended either of the past two follow-ups (at 22 and 27 years).

**Results:**

There were considerable differences between active and inactive participants, with active participants perceiving far more personal and collective benefits from their participation. Inactive participants described being constrained by structural barriers around work and life, whereas active participants were able to overcome them to attend follow-ups. Inactive participants also described the value of extrinsic incentives which might motivate their attendance, and active participants described the role of their parents as significant influencers in their propensity to remain in the study.

**Conclusions:**

This paper provides rich descriptions of what participation in a long-running study means to participants. Use of a social marketing framework ensured that participants were constructed as ‘human consumers’ who are influenced by individual and broader social systems. Understanding participants in this way means that differentiated strategies can be tailored to enhance retention.

## Background

Longitudinal cohort studies are a critical resource for medical research, enabling discoveries not possible with other study designs. The collection of information related to the same individuals at multiple time points enables researchers to understand the life-course of health and disease and the temporal relationships with factors influencing that life-course. This unique understanding contributes to insights into mechanisms, and informs when to intervene and what to target in interventions to enhance health and prevent or ameliorate disease and its sequelae [1, 2].

Successful longitudinal cohort studies are challenging to establish and potentially even more challenging to maintain [3]. It is usually difficult to secure long-term funding for longitudinal studies, and setting up and maintaining a longitudinal cohort study is costly [2]. Establishing and maintaining good governance is also often a challenge [3]. However, the challenge most commonly reported is that of maintaining adequate participant involvement.

The ongoing value of a longitudinal cohort study is to a large extent dependant on its ability to retain a sufficient proportion of participants, who are representative of all participants, for ongoing assessments. A decline in the retention rate over time is typical, for example when participants move or lose interest [4, 5], but results in a reduction in power [5, 6]. Further, differences between those who ‘drop-out’ of a study and those who are retained may bias the findings [5–8]. It is therefore not surprising that the importance of keeping retention rates in longitudinal cohort studies as high and unbiased as possible is widely reported (see for example [2, 4, 5, 9]). It is also common practice in cohort profile papers to report retention or attrition rates, along with a comparison of specific characteristics of those who remained in the cohort with those who were lost to follow-up, to examine potential bias [10–18].

Whilst the challenge of maintaining high and unbiased retention rates is widely recognised, along with a body of research examining strategies used to enhance retention [9, 19, 20], few studies have focussed on the importance of understanding the participant experience underlying retention. Of the latter, most have focused on ‘sick’ [21] rather than ‘healthy’ cohorts [22].

Other successful longitudinal studies of the general population, such as the Framingham Study which has been running for over 70 years (https://www.framinghamheartstudy.org/fhs-about/history/), have been able to maintain sufficient, unbiased retention. Understanding how participants experience their involvement in longstanding studies like this is likely to reveal deep information about the mechanisms which drive retention.

The aim of the current study was to provide unique insights into the participant experience underpinning retention in a longitudinal cohort by utilising a social marketing approach, whereby participants are constructed as consumers, through the co-created experience of being research participants as opposed to research subjects. The paper unpacks what marketers refer to as the ‘understanding’ phase of a social marketing approach [23]. In our study, this involved determining what benefits cohort participants derive from volunteering their time; what barriers they face; what might motivate them to attend; and who influences their propensity to return. Competition also forms part of the understanding phase in social marketing, but it was excluded because it generally refers to behaviours which potentially compete with the desired behaviour (i.e. attending Raine Study follow-up studies every few years). Given the infrequent nature of follow-ups, understanding barriers was a more appropriate indication of the types of issues that participants face in committing to attend follow-ups, rather than any competition per se.

Based on a deeper understanding of these parameters, a second companion paper (under review) addresses issues that relate to the ‘4Ps’ of social marketing: providing insights about what cohort participants are ‘buying’ (or exchanging) in terms of a desirable Product; the monetary and non-monetary costs associated with participation (the Price they pay); the importance of the Place in which participants attend study activities; and any communication issues that persuade them (Promotion) to come back. Understanding the benefits, barriers, motivators and influencers, however, is a necessary first step in a social marketing approach, and is therefore the focus of this first paper.

Using a marketing framework as the theoretical anchor in this investigation is beneficial, primarily because it can inform the creation of ‘satisfying exchanges’ that provide value for consumers [24], which in essence means giving them what they need and want. Donovan and Henley, two leading social marketing authors, assert [25] that unlike mainstream or commercial marketing, social marketing is premised upon a different end goal: one which aims to bring about health and social benefits for populations rather than increased profits for corporations. Given longitudinal studies are designed to provide future population benefits in the form of scientific knowledge and health innovations, a social marketing framework qualifies as a fitting approach for this investigation. The idea that cohort participants exchange their time and energy to achieve external benefits – in much the same way as other people do when they give blood or take the time to sort their recycling – is also an important factor that social marketing strategies can help to leverage. Determining whether or not participation is ‘worth it’ for participants (i.e. that it is a ‘satisfying’ exchange) is what this study aimed to investigate.

Reflecting this social marketing framework, this investigation set out to facilitate a comprehensive understanding of factors contributing to retention and attrition of the Generation 2 participants in the Raine Study, a longitudinal cohort study that has been running in Western Australia for the past 30 years. This first paper addresses the research question: What are the specific barriers, benefits, motivators and influencers associated with attending Raine Study follow-ups?

## Methods

This research aimed to gather rich narratives about the experiences of Raine Study Generation 2 participants, particularly as they moved into their late 20s – a time where life tends to become more complicated. The following section provides a brief overview of the Raine Study, as context for this research.

### The Raine study

The Raine Study is a longitudinal cohort study running in Western Australia since 1989. The initial aim of the study was:

to develop a large cohort of Western Australian children studied from 18 weeks’ gestation to ascertain the relative contributions of familial risk factors, fetal growth, placental development and environmental insults to outcomes in infancy and to the precursors of adult morbidity [17].

The Raine Study has subsequently developed conceptually into a “life-course framework”, which aims to understand the multiple interacting domains including genetics, phenotypes, behaviours, physical environments, and social outcomes such as education and work [17]. Through the publication of over 500 scientific papers, it has made a significant contribution to understandings of the life-course of many health and disease issues, along with contributing factors and consequences stretching from early in utero life through to adulthood [17].

Initial recruitment of 2900 women (‘Generation 1’) who were 16–20 weeks pregnant between May 1989 and November 1991 resulted in the cohort of 2868 live births (‘Generation 2’). Generation 1 (parents) was asked to bring their children (Generation 2) in for assessments and to complete questionnaires as part of the Raine Study at 1, 2, 3, 5, 8, 10, 14 and 17 years of age. Generation 2 participants were subsequently invited independently for assessments and to complete questionnaires when they were 20, 22 and 27 years old. These assessments are known as follow-ups and involve regular biomedical testing, which varies in the degree of invasiveness depending on the aim and scope of health issues being investigated. Assessments commonly include non-invasive testing such as measurements of height, weight and blood pressure, through to more demanding testing such as fitness testing to invasive assessments including taking blood and urine samples. A full list of assessments undertaken at the various follow-ups is provided in the published cohort profile paper [17].

In order to encourage attendance at these follow-up studies, Raine Study staff seek to communicate with participants across a number of different platforms which have been deemed successful as they have entered into adulthood; this is primarily through social media, electronic newsletters sent to all participants and personalised emails, text messages and phone calls. Unsurprisingly, some attrition has occurred across the childhood, adolescent, and young adult life stages (see [17]), though analysis of characteristics of those lost to follow-up suggests minimal bias in retention [6].

### Sample and recruitment

Recruitment for this qualitative study was undertaken by the Raine Study Follow-up Manager, via telephone or email, with the aim of recruiting both ‘active’ and ‘inactive’ participants. ‘Active’ participants were defined as those who had agreed to attend their 27 year follow-up appointment, while ‘inactive’ participants were defined as those who had not attended either of the past two follow-ups (22 and 27 years); they may have, however, attended a previous follow-up at 20 years or earlier.

### Data collection

While the initial aim had been to conduct a mix of focus groups and individual interviews, the research team revised the data collection strategy after one focus group was conducted, and instead used individual interviews to collect all subsequent data. This was due in part to difficulties in coordinating participants for focus groups, and also because participants in this first focus group suggested that inactive participants may not be willing to discuss, within a focus group setting, why they had dropped out of the study.

A semi-structured interview guide (see Additional file 1) was drafted, with the goal of developing an understanding of what it is like to be a Raine Study participant. Participants were encouraged to explain what their experiences had been so far, focusing on their recall of participating in the Raine Study at different life stages, and what their expectations might be as they mature. In keeping with the social marketing theoretical framework underpinning this research, questions were specifically framed to investigate the barriers, benefits, motivators, and others who might influence their propensity to attend follow-up studies.

All participants provided written or verbal consent before the interview commenced. In order to minimise social desirability bias, especially from active participants, the two lead authors who conducted the interviews made it explicitly clear that they were neither employed by, nor were they contracted by the Raine Study to conduct the research. Participants were encouraged to ‘have their say’ and were assured that their responses would be deidentified, and therefore could not be identified by the Raine Study staff. That is, the authors spent considerable time building rapport before commencing the interview by reinforcing the intent of the study: to understand participants’ real, ‘behind the scenes’ experiences; ones which they probably never had the opportunity to express previously.

This type of rapport-building technique forms part of a larger suite of techniques designed to ensure trustworthiness in qualitative research [26]. Other techniques to aid rigour and trustworthiness [27] also guided this research. For example, the interview guide was constructed and reviewed by members of the research team and then by a panel of experts associated with the Raine Study. Saturation of the data was reached when interviews with both active and inactive participants did not reveal any new insights. This was able to be achieved relatively quickly given the demographic cohesiveness of the sample [28]. Early on, however, the recruitment process was rendering more female than male participants. To ameliorate this, the Raine Study staff were asked to target male participants (particularly inactive ones) so that saturation could be reliably judged.

The interviews took place between September 2017 and February 2018 and each lasted between 20 and 90 min; they were digitally recorded and transcribed verbatim. The focus group and interviews with active participants took place at the research facility where recent follow-up visits have occurred (Raine Study House), while interviews with inactive participants were conducted via telephone. 

### Data analysis

Guided by Braun and Clarke [29], each transcript was read twice before analysis to determine the tone, patterns and general themes amongst the respondents [29, 30]. The first author conducted initial inductive coding of transcripts, using NVivo 12. During this process, constant comparative data analysis was conducted, whereby as transcripts were examined, meaningful words and phrases [30] were classified into ‘open’ codes, using either descriptive or in vivo terms. These were then grouped deductively into broader axial codes which were pre-determined by the social marketing dimensions used to underpin this stage of the research, namely: benefits, barriers, motivators and influencers. At this point, the second author reviewed the codes, and then consulted with the first author to resolve inconsistencies and “eliminate errors and misinterpretations of the perspectives” [31]. While an inter-rater reliability (IRR) score was not considered appropriate for this research, primarily because coding performed by the second author was intended to highlight disagreement or alternative interpretation [32], coding reliability was established through loop-like discussions between the coders, where open codes were challenged and refined. This meant that some of the open codes were re-organised and re-assigned to a different dimension in the social marketing framework being applied to the data. Ultimately, this provided a robust and rigorous structure from which to construct the narrative in the paper. It also allowed for each of the corresponding themes embedded in the social marketing dimensions to be unpacked, providing a deep understanding of the issues which seemed to affect active and inactive participants’ propensity to attend Raine Study follow-ups.

### Ethics

The research received ethical approval from Edith Cowan University’s Human Research Ethics Committee in September 2015 (Approval number 18242). All identifiable data were removed during analysis, including names of people or places, to maintain participant anonymity. In this paper, participants have been assigned pseudonyms and identified as active or inactive.

## Results

In total, 29 Generation 2 participants completed either a focus group (*n* = 3, all female) or individual interview (*n* = 26). Of these, 17 were active participants (11 female), and 12 were inactive (6 female). Inactive participants had not participated in follow-up appointments for at least 5 years (mean 10.7 years, range 5 to 22 years).

The specific themes which aid an understanding of both active and inactive Raine Study participant experiences - benefits, barriers, motivators and influential others - are discussed next.

### Benefits

Reported benefits of participating in the Raine Study included both personal benefits and collective benefits, but the perception of these benefits differed between active and inactive participants. The themes are listed in Table 1 and then interwoven in the narrative below.

**Table 1:** Personal and collective benefits

Theme	Subtheme	Codes	
		***Active participants***	***Non-active participants***
**Benefits**	Personal benefits	Early detection	Health check-ups
		Health check-ups	
		Assuredness/Peace of mind	
		Benchmarking/self-monitoring	
		Immediacy of results	
		Self-preservation/actualisation	
	Collective benefits	Collective-outcome efficacy	
		The ‘greater good’	

An important benefit identified by some of the active participants was the early detection of personal health issues through Raine Study testing. While these health issues ranged in severity and included conditions such as colour blindness and glue ear through to pancreatic cysts, participants valued that they were discovered early. Some active participants who had experienced more serious illnesses explained how they might have suffered for longer, or later in life, had it not been for their participation in the Raine Study:

*…the payoff is pretty brilliant. So I know things about myself that I would not have known until maybe an adult.…I think the 15 year follow-up - when they did fertility and they did ultrasounds of our ovaries - I found out that I had polycystic ovarian syndrome … that's something that I potentially wouldn't have found out until I was in my 30s even because I would have gone on birth control, it would have masked the symptoms….I would have been wondering why I put on weight so easily, I would have been wondering why I grow body hair there but I never really would have made the connection … presumably if I wanted to have kids and then been hit by the news that ‘actually you might have fertility problems. Actually, you've got a tonne of cysts’. And that was so valuable, so that's probably the biggest impact on my life is finding out things about myself that I just wouldn't have known. I would have been in the dark. (Renae, active)*

Finding out things about themselves, that they otherwise would not have known, was highly valued by all of the active participants, even those who had not discovered any early illness or health condition. They inherently understood that being involved with the Raine Study provides them with regular check-ups where they can potentially screen health conditions:

*...this is a weird way to describe it, but there is a part of me that likes the fact that I guess I'm being scanned or I'm getting blood tests so they're checking my asthma and if there was something wrong with me it might have been picked up. Maybe I haven't been walking around with a tumour or something for five years and not known about it because they do blood tests […] So it's like a health check every few years which I guess is reassuring… (Cassie, active)*

This idea of assuredness through regular testing was considered a key benefit by active participants, one which Renae described as “empowering” in terms of providing her with a sense of “control”. As well as the potential for early detection, receiving medical information about their health status was also described as beneficial by active participants. This provided a peace of mind or a feeling of relief, at least when their results revealed no significant health issues. Again, for Renae, this seemed to support positive mental health:

*It's rewarding to feel like you're ahead, you're a step ahead … For someone that has anxiety a lot of the time I prefer knowing something. I hate the unknown … so to be able to have that [screening] is quite good. (Renae, active)*

Renae’s reference to being a “step ahead” suggests that some participants benchmark their own health, providing a core benefit to their participation. That this was “rewarding” implies a positive payoff as a Raine Study participant. Other active participants described how they are able to self-monitor and gauge their health over time. They particularly appreciated the information provided from DEXA scans and other ‘big picture’ tests, giving them the opportunity to see improvements or deteriorations. Some active participants recalled how they particularly enjoyed this benchmarking as a child, seeing, for example, how far they could jump compared to other children or in comparison to their last visit. Now, in their late 20s, most of the active participants described an interest in gauging how they were changing as they were getting older. This was an important benchmarking benefit that they said would keep them coming back.

As well as appreciating benchmarking, active participants also valued the immediacy of receiving medical information from the Raine Study:

*…last time they were looking for cancers and things like that and so obviously I find that beneficial in that okay well I'm free for now, it's good. Things like, they were looking at my eyes today, so they can say well your eyes aren't very damaged from the sun […]. So it is relieving because I think most people have concerns about their health, about what they can and can't do, how much protection they need for this and that. And so to just be clarified on where you stand it's very helpful, I think. […] and also too - as an immediate thing because they find out things and they might say well you have a cancer, you have this or that, you need glasses, anything. And so that's valuable for anybody really. (Guy, active)*

Active participants perceived that this type of Raine Study testing gave them a unique benefit which set them apart from the rest of the population. They often referred to GPs and mainstream medical services, to contrast what they received from the Raine Study. Kimberley, for example, noted that GPs “wouldn’t do half of this stuff”, explaining that by the time one might actually need treatment it would likely be “too late”. The test results that Raine Study participants accumulate at each Raine Study follow-up forms a medical repository which can be drawn upon as a rich information source now and in the future, and thus provides a tangible resource for self-preservation and self-actualisation.

In contrast to active participants, inactive participants did not describe personal benefits with the same amount of enthusiasm and detail. While a few were able to recall how the tests that they did when they were younger revealed useful information for them, the barriers to participation – which are described in more depth later - seemed to outweigh any potential benefit:

*..I’m probably not getting any gain from it. So that’s time that I could use to spend with my kids or doing other stuff. (Chris, inactive)*

Significantly, Sam was the only inactive participant who alluded to a potential benefit by describing the Raine Study as “an opportunity of a lifetime”. As most of the inactive participants were already at the point at which they perceived the barriers outweighed any potential benefits, Sam’s insights are somewhat unique. As he explained, he could not access “all those types of checks in hospitals”, which is in keeping with the benefits appreciated by active participants. However, when prompted for more information about the types of checks he was referring to, he was not able to elaborate. Instead, he reframed his answer in an apologetic tone, repeating several times that he did as much as he could for the study. While Sam had not attended a follow-up for a few years, he put this down to the ‘busyness’ of work and life – a barrier identified by many other inactive participants, and discussed later in this paper. As Sam explained:

*Like where I’ve been busy with work and stuff where I haven’t been able to catch up with you guys but most of the time I’ve tried my best to obviously work it out. Yeah. (Sam, inactive)*

As a projective technique, Sam was asked what he might say to other Raine Study participants if they were thinking of withdrawing. His response indicates that he still considers himself a part of the Raine Study:

*Mine would just be like why quit now? You know you’ve done it this far, why wouldn’t you go the whole – like obviously you’ve studied like as a person for this long, why wouldn’t you continue it on? Like that would be more the question I would ask them when I see the answer. Obviously you know if they had family issues and bits and pieces then fair enough, but. (Sam, inactive)*

While it is possible that Sam may have been influenced by social desirability bias, it is also possible that he and other participants who have only recently failed to attend a follow-up intend to return and remain as active participants. In this case, finding ways to reduce the barriers which prevent recently inactive participants from attending future follow-ups is critical.

Apart from a greater appreciation of the personal benefits amongst active participants, there were also marked differences in how active and inactive participants described the collective benefits attributed to others through their voluntary contributions to medical research. Active participants expressed a deep conviction about these collective benefits:

*It’s about making an impact, it’s something really simple and it’s not an imposition in my life but it’s going towards research which I know there’s not many around the world which is so longstanding […]. As simple as taking my blood or doing the scan, it’s really nothing for me but it makes a difference. I’m so small that nothing – my part, some blood, some surveys and scans – is really not imposing but in the bigger picture it makes a difference. And not just here, throughout the world and not just now, it’s like future as well. (Erin, active)*

Importantly, all the active participants described how their efforts to attend Raine Study follow-ups were really “nothing” compared to the benefits which would accrue to the broader population in the future. For Adrian, this was a good way to contribute to something really valuable without having to “save the world”, presumably all on his own. Erin’s comment above, about being “so small”, suggests she perceives that her own efforts make a difference when they are viewed collectively, as part of the bigger picture. The theoretical concept of ‘collective outcome efficacy’ [33, 34] is particularly relevant here, in that the active participants seemed to believe that their individual contributions as Raine Study participants would ‘add up’ to make a difference to the health of others now and in the future. This is strongly illustrated in the comment below, where Alison described a conversation with her mother about the value of the Raine Study. As she perceived it, this was the lightbulb moment that cemented her understanding of the collective benefit that the Raine Study enables. She recounted her mother’s words, and her reaction, from that conversation:

*You're giving so much data to people that they're going to be able to find out so much with what you're providing with simple tests that you're doing. It takes a few hours out of your day but with everyone, all the participants that are in it it's actually providing so much data that's going to provide so much knowledge for people so she gave me a much broader understanding. And then I was like ‘oh my gosh, I'm giving to the greater good in a way. I'd never thought of it like that’. (Alison, active)*

It is possible that active participants have a strong sense of collective outcome efficacy because they, like Alison, understood and internalised concepts about the greater good and humanity as they came of age in the Raine Study. The intrinsic rewards that often ensue from altruistic behaviour were described by a number of the other active participants, but is best exemplified in Erin’s comment below:

*Through my experience as a kid and the perspective of a kid and as you're getting older your understanding of it [the Raine Study] and the difference it makes, like how it makes you feel, like you feel good to be [able] to contribute (Erin, active)*

Conversely, although some of the inactive participants also described how their contributions to the Raine Study might make a difference - especially in terms of helping future kids – their narratives did not demonstrate a collective confidence that what they were doing would make a compelling difference for others in the future. Inactive participants could not explain how their data from previous studies might be used or what it had been used for thus far, and indeed, ‘helping future kids’ seemed to be given almost as a token answer. Their response could be interpreted as evidence of social desirability bias, and this is discussed further in the discussion section.

In saying that, even the active participants were unsure of the impact that the Raine Study had made in terms of medical research, although they were able to cite some research outputs they had been told about “somehow”. They were, however, adamant that it *was* doing good and that it would *keep* doing good if “we all keep going”. Thus they did not need tangible evidence of the study’s impacts to believe in the value of their contribution to the greater good. In contrast, most of the inactive participants only mentioned collective benefits when they had a personal experience that ‘opened their eyes’ somewhat to the need for cohort studies like the Raine Study. In most cases this was because a child, parent or sibling had been diagnosed with a chronic illness.

An intrinsic sense of collective outcome efficacy seems to augment the value active participants receive from their participation. For example, while Cassie and Alison both described barriers such as lack of time and inconvenience, they were adamant that it was the collective benefit that kept them coming back:

*Really, if you don't understand the bigger picture of what they're trying to achieve. Our time's precious, you wouldn't - or I wouldn't [do it]! I guess that's the only thing [the collective benefit], because why else would you do it? (Cassie, active)*

*I don't think anyone particularly likes the sleep study, but if it's one night I think that there was quite a bit of information that was gained from that hopefully. If you can't - you can put it in perspective quite easily, just be like ‘oh it was one or two nights’ if you did the follow-up, a couple of nights. Then you can be like, ‘well’ there's so much to gain from that, hopefully. (Alison, active)*

While inactive participants also mentioned some collective benefits, they were more likely to perceive structural barriers such as transport difficulties that prevented them from attending. Such structural barriers may also have served to dilute their stronger convictions in relation to the greater good. If this is the case, it would make sense to leverage and reinforce collective outcome efficacy for active participants, but not necessarily for inactive participants. They, it would seem, need more tangible solutions to the barriers they face, which are often highly complex and difficult to change at an individual level. Barriers for both the active and inactive participants are described next.

### Barriers

The Generation 2 participants we interviewed cited barriers that are commonly understood in public health as social determinants: those social forces (at the individual and structural level) that influence why people do (or do not do) certain things. In this regard, the choices people make are often influenced or constrained by factors which are beyond their control. This is true even when they have positive intentions to do something which they might consider good, healthy or worthwhile. In our study, behavioural intention relates to a participant’s propensity to attend follow-ups. The themes identified for individual and structural barriers are tabulated below (Table 2) and the differences between active and inactive participants are then described in the following section.

**Table 2:** Individual and structural barriers

Theme	Subtheme	Codes	
		***Active participants***	***Non-active participants***
**Barriers**	Individual constraints	‘No skin off their backs’ (some family pressures)	‘Life gets in the way’ Family and health challenges
	Structural constraints	(some work pressures)	Work and economic constraints
	Knowledge and awareness	N/A	Knowledge gaps about Raine Study processes

Participants, particularly the active ones, were, on the whole, positive about the benefits accruing to them and others through their voluntary involvement in the Raine Study research. They did, however, describe how work and family commitments made it “sometimes” difficult to attend a follow-up appointment.

Commuting to the Raine Study House for clinical assessment was cited as a difficulty, as was finding the time when life ‘gets in the way’. Active participants, however, were more likely to overcome these difficulties, and were less restrained by the structural constraints than inactive participants. Most of the active participants shrugged off the inconveniences of taking time off work and juggling their social calendars, by declaring that attending follow-ups required no “skin off [their] backs”:

*Yeah, the only thing you have to do is set aside the time and that’s it. And when you’re volunteering to give up a bit of blood or go through a test, it’s not depriving you of anything, really. …it’s not like I’m sacrificing anything for it. (Adrian, active)*

*What's a couple of hours for me on a Saturday compared to what can be achieved through everything that they've got so far? (Cassie, active)*

Cassie’s comment implies that the time she sets aside to attend the odd follow-up on a weekend - which is often sacred time for many Australians – is worth the effort, primarily because the Raine Study has come “so far”. This suggests that Cassie values the social ‘good’ of medical research and what it can achieve, and privileges these perceived benefits over any inconvenience incurred through her attendance at follow-up appointments. Pia was somewhat more candid about the effort involved in commuting to Raine Study House, but, like Cassie, her belief in the collective benefits ensuing from her participation (similar to collective outcomes efficacy described earlier) appeared to outweigh perceived barriers, and underpinned her commitment to participate:

*It's too hard to get here, to make the time when you're working full-time to make the time to get here. It's quite tricky to get to if you don't have a car or you live quite far away. So, if it's something you're not passionate about, if you're not super passionate about your results or you're not passionate about making the world a better place [you wouldn’t attend]. (Pia, active)*

Both Cassie and Pia described themselves as professional women, having graduated with a university degree and secured fulltime employment. They, along with most of the active participants, described their lives as busy but uncomplicated, having avoided, so far at least, too many personal hardships. Renae described herself as somewhat “unique”, but acknowledged that life may not always stay that way:

*I'm probably unique […] I don't have any kids, I'm not married, I live a fairly independent life. Maybe in a few years it will become a little more difficult [to attend] but right now it's really fine. (Renae, active)*

These perspectives serve to juxtapose some of the barriers described by inactive participants. While inactive participants also expressed positive sentiments about the Raine Study – albeit with less conviction – they apologetically explained how certain social forces made it virtually impossible for them to attend:

*I’ve got two kids of my own and another one on the way, I work fulltime and so to put aside time to do that I’d – I know, it sounds selfish… (Chris, inactive)*

While inactive participants described economic hardships which made taking time off work difficult, and which were further compounded by transport and parking costs, some also described significant health challenges which prevented them from attending. Ricky explained how personal health issues meant he had spent a lot of time in surgeries and hospitals. For him, having to attend the Raine Study appointments perpetuated this experience, and meant that he chose not to attend follow-ups for his own mental well-being. Similarly, Michaela shared a fairly detailed story about her very young son being diagnosed with a terminal illness. She explained that she couldn’t commit to Raine Study follow-ups, now or in the foreseeable future, because it was “just too much” to bear in the face of her son’s treatment regimen.

Of interest here is that while illnesses or health conditions were identified as significant barriers by inactive participants, such challenges seemed instead to motivate some of the active participants to remain engaged with the Raine Study. As previously discussed, active participants experiencing a personal or family health issue perceived their involvement with the Raine Study as offering unique benefits, rather than being a burden. For inactive participants, therefore, it is critically important to find ways to address the structural barriers, or to provide other benefits or incentives which would outweigh them.

Another potential barrier was a lack of awareness of the Raine Study processes. For example, one participant who had only just reinstated contact with the Raine Study after a period of not attending follow-up appointments, explained how she had thought the Raine Study had ended. She was somewhat surprised to find out that it was still running and that the Raine Study staff had been trying to locate her. A comment by another inactive participant suggests a similar knowledge gap about the longevity of the Raine Study:

*…it starts to get hectic I think, but yeah I don't know. I don't know what they could do really, yeah. I just - yeah, I don't know. How long does the study go for now, until they're 30 or? (Richard, inactive)*

Richard’s lack of knowledge also seemed to extend to other aspects of the Raine Study, as the following comment suggests:

*They don't do the studies on the weekends. I don't have time during the week. I can't just take time off work to do it. That's the only reason why I haven't participated; it’s because of adult life pretty much. (Richard, inactive)*

Although follow-ups are conducted on weekends, comments like this indicated that there may be limited awareness of the study processes and other knowledge gaps across the cohort. Potentially then, some inactive participants might be more motivated to attend if they had a better understanding of the Raine Study, and knew about alternative testing times (already) offered. If knowledge gaps like these are apparent, they can often be addressed through social marketing strategies, which are discussed in the second paper.

### Motivators

In keeping with the social marketing approach, participants were asked if there was anything that the Raine Study could ‘say, show, give, or do’ for them which might improve their experience as study participants. This type of prompting technique is discussed in most social marketing handbooks, including Lee and Kotler’s 2016 text [23]. These authors note that motivators are different to benefits (described earlier) in that they constitute the ideas provided by participants; ones which would “make it more likely that they would adopt the desired behaviour” [23]. Table 3 lists the themes identified for motivating factors.

**Table 3:** Intrinsic and extrinsic incentives

Theme	Subtheme	Codes	
		***Active participants***	***Non-active participants***
**Motivators**	Intrinsic incentives	Professional recognition	N/A
	Extrinsic incentives	N/A	Practical incentives telehealth

When participants were asked if they could think of any additional motivators that might incentivise their ongoing commitment to the study, they found it difficult to generate ideas. In hindsight, this type of format would have been better facilitated through a focus group. However, some of the inactive participants suggested that more practical incentives – for example fuel and childcare - might help them to overcome structural barriers such as transport costs. The fact that ‘life gets in the way’ for these participants prompted them to suggest that the Raine Study could implement some sort of telehealth service, or that Raine Study staff could come to them in their own homes. Others suggested hub-like centres, where Raine Study data collection could be undertaken in hospitals or other local health services. A number of the active participants discussed how their participation provided some professional recognition – sitting on Raine Study advisory groups for example. Three of the active female participants thought that this could be taken further, perhaps by providing certificates or ‘additions’ for their resumes.

Interestingly, most active and inactive participants rejected the idea that financial incentives like vouchers – for movies or entertainment – would encourage them to attend, especially because they recognised that the research was already costly and this would only add to those costs.

### Influencers

As well as highlighting motivators, the interview data also revealed other influencing factors (Table 4) which provides some additional insights which might influence participant retention.

**Table 4:** Influential factors

Theme	Subtheme	Codes	
		***Active participants***	***Non-active participants***
**Influencers**	No choice as a child	Positive transition from child to adult	N/A
	Parents	Positive parental attitudes / family commitment	N/A

For example, when participants were asked about their parents’ involvement in the Raine Study, both active and inactive participants reverted to discussions which described how their parents had essentially signed them up, acknowledging that they had no choice in the matter. That is, as infants they simply went where their parents took them, including for Raine Study testing. Participants recalled how, as children, they didn’t really understand the study, but were aware they *had* to attend every few years, which often meant being treated to a rare day off from school. As they reflected, it wasn’t until they became teenagers that things began to change. Most participants described being somewhat reluctant to attend as a teenager. Some of the active participants clarified this by asserting that this was not because they were disgruntled by the testing, but because they were disgruntled by having to do anything they were told to do at that age:

*So when I was little I was not concerned at all, I just came along because my mum brought me and so they did tests and I really had no idea. Then when I was a teenager I was not so keen on it because I felt I didn't want to do the questionnaires, I didn't want to do any of it really as probably most teenagers are, I was quite anti-people making me do things. So then as a teenager […] I wasn't so into it. But now I'm happy to do it. I think it's great. (Guy, active)*

The transition from being somewhat disgruntled (as an adolescent) to happy (as an adult) was not expressed by inactive participants. Inactive participants were more likely to emphasise how they had no say in their parents’ decision to enrol them in the study:

*I guess I didn’t really know much about what – I didn’t really know what it was for or what it was about or why I was going other than my parents said it was important that I went so when my parents stopped being able to say to me that it was really important that I went - I stopped going, if you know what I mean? (Chris, inactive)*

Another key difference between active and inactive participants was the way they described their parents’ attitudes to the Raine Study. For example, active participants emphasised the involvement of their parents as important positive influencers in their decision to stay on as adult participants. Guy added to his comment earlier by asserting that he might have dropped out if it were not for his Mum. In fact, all the active participants described how their parents would be very disappointed in them if they dropped out now. Maree went on to explain how her Mum instilled the value of the study in her, while Renae reported on her parents’ commitment to the study:

*I just like the principle behind it and I suppose my mum went because she was the one that obviously originally signed up for it. […] My mum's philosophy of the fact that the research is contributing to improving other people's lives essentially and I think that's what she's always instilled within us, is that it's really important to be able to - if you've got this opportunity to give it - all the honesty and everything that you can give it, just to make sure that the tests […] that they can use this research for good. […] and their beliefs [her parents] that they have, I have, which then relays onto why I stay in it. (Maree, active)*

*I think if my parents were like, oh, far out, I have to take you to this study now and if they were negative about it or if they were, oh I'm sick of having to do all this then I think I probably would have quit. I think I'm very influenced by them as well. They got so excited about doing the sleep study, they love taking part. (Renae, active)*

Inactive participants did not reveal as much about their parents as the active participants, other than simple reflections about how their parents told them the study was important. Nor did they necessarily know how their parents would react if they withdrew from the study altogether:

*Yeah, I’d say to be honest if I did drop out you know I’d say she would – she may be – might be a little bit upset, she might not be but it’s, I would say yeah like obviously you know you can only help as much as you can before I guess it takes too much. (Sam, inactive)*

It seems that for the active participants, the Raine Study was viewed as a family commitment, even after the participants became independent. In contrast, inactive participants’ families may not have embraced the Raine Study with the same enthusiasm – it may have been perceived as an individual commitment, and so once the child became a young adult, the parents felt no obligation to encourage them to continue to attend. That is, parents’ ‘buy in’ may be critical to retention, even after participants become adults. That is not to say that parents are to blame here; it is likely that they too may have been constrained or pre-occupied with those other barriers described earlier. This was best exemplified when Chris couldn’t really remember if his father – as a single parent – was particularly committed to the study, primarily because he had a “full plate” looking after other children and working hard.

## Discussion

This paper presents the findings of an innovative study that applied a social marketing framework to explore attrition, retention and engagement of Generation 2 participants in the Raine Study. In doing so, it provides a unique perspective on the experiences of participants in longitudinal cohort studies. While a number of papers have reported strategies to enhance retention in longitudinal cohort studies (see for example [9, 14, 19, 20]), the approach adopted in our research facilitates a broader understanding of what participation in a long-running longitudinal cohort study *means* to participants. In particular, the social marketing framework draws on a socio-ecological model [35] that positions individuals within broader social systems and networks. Within this model, an individual’s choices and behaviours are perceived as influenced by a complex range of individual and population level factors (social determinants). This is significant, given that attrition in the Raine Study cohort has been found to be slightly greater amongst participants reflecting socioeconomic disadvantage [6]. In this context, retention strategies such as maintaining regular contact with participants, routinely disseminating findings, and promoting rapport between the research team and participants (see for example [14]), that are typically used to maintain interest and engagement, may not be enough to assist participants overcome structural barriers such as low income, lack of transport or limited time to attend appointments due to family and/or work commitments.

There is, however, some evidence in the literature of additional retention strategies that reflect an awareness of broader challenges which participants may experience. For example, Clough et al. acknowledged that the women participating in their research who had experienced domestic violence had “multiple and complex factors in their lives that make retention in a longitudinal research study challenging” [36]. One strategy employed in their study was the provision of childcare during follow-up appointments [36]. Notably, childcare was also a motivator mentioned by some of the inactive participants in our study. Other studies have reimbursed parking and transport costs, while some offered financial compensation for follow-up attendance [9]. Interestingly, in our study both active and inactive participants rejected the idea of financial incentives to motivate attendance, observing this would have a negative impact on the study. This finding contradicts the results reported in Booker et al.’s 2011 systematic review [19] and should be further investigated in the Raine Study cohort. Still, it does suggest that even where a participant has dropped out, they still retain a level of goodwill towards the study.

It may also be true that this goodwill, manifested through the medical value that is afforded from research such as the Raine Study, is somewhat overstated and obligatory. That is, participants may have responded in socially desirable ways because they inherently know that the Raine Study produces benefits for current and future generations. While this may be a limitation in this study, active participants seemed genuinely proud of the Raine Study and were enthusiastic about saying positive things about it. Inactive participants, however, tended to provide ‘token’ statements about the Raine Study’s value for future generations. Of course, the barriers to participation were less constraining for active participants compared to inactive participants; hence, it is more likely that their positive responses about the collective benefits of the study were authentic compared to some of the positive sentiments expressed by inactive participants. For inactive participants, the barriers ultimately limited their ability to contribute, no matter how inherently good they reported the study to be.

Further limitations in this study may be reflected in the parameters which were established to denote whether a participant was considered active or inactive. In some cases, if participants had not attended the last study (inactive), but still considered themselves a part of the study, there may be more distinct differences in comparison to inactive participants who have no intention of returning to the study. It would be useful to conduct further research which segments active and inactive participants into more specific categories, in order to detect differences in the themes which have been presented in this paper.

## Conclusion

The findings presented in this paper extend understandings of factors that influence attrition and retention in longitudinal cohort studies. Using a social marketing lens, participants in the Raine Study were conceptualised as consumers of the research experience who provide access to their personal data for medical research, in exchange for personal and collective benefits. In our research, the willingness and capacity for individual participants to remain in the study appeared to be influenced by a range of personal and collective benefits, such as regular check-ups and a belief they were helping others, and barriers, including transport costs and inconvenience. Significantly, inactive participants were more likely to perceive that the barriers they experienced outweighed any benefits they received, while the reverse was largely true for active participants.

Our research also highlighted additional motivators that might encourage retention, particularly amongst inactive participants. For example, some inactive participants suggested that practical incentives like fuel vouchers would help them, while others indicated that being able to attend Raine Study appointments at local health centres would be more convenient. These motivators may serve to reduce the impact of structural barriers.

Finally, data analysis revealed the important role that Generation 1 parents play in shaping their children’s attitudes towards participation in the Raine Study, and in motivating them to continue their involvement once they turned 18. In this context, parents could be conceptualised as influencers, helping to motivate (or not) their young adult children. This is a particularly important finding, as it suggests that even after participants become independent, parents continue to influence retention. Indeed, our research suggests that parents’ ‘buy in’ may be crucial to the ongoing success of pregnancy or birth cohort studies such as the Raine Study, and thus strategies that reinforce and reconnect parents throughout the life of such studies should be investigated in future research.

In the second paper of this two-part series (under review), we extend our application of social marketing by using the 4Ps (Product, Price, Place and Promotion) framework to explore how Raine Study participants currently experience these elements. This strategy enabled us to highlight where strengths and weaknesses in Raine Study processes might exist, or where opportunities to augment these processes could add value. In this context, a social marketing framework offers a novel approach to leverage the benefits and minimise barriers discussed in this first paper - and thus support retention. Such an approach is potentially transferable to other longitudinal cohort studies keen to maximise retention.

## Supplementary information

**Additional file 1.**

## Data Availability

The datasets generated and analysed during the current study are not publicly available due confidentiality requirements specified by Edith Cowan University’s Human Research Ethics Committee.

## Abbreviation

4Ps: refers to Product, Price, Place and Promotion in marketing theory
