# Supplementary material for: Informing retention in longitudinal cohort studies through a social marketing lens: Raine Study Generation 2 participants’ perspectives on benefits and barriers to participation
Source: BMC Med Res Methodol. 2020 Jul 29;20:202. doi: 10.1186/s12874-020-01074-z (PMC7389450; doi:10.1186/s12874-020-01074-z)
Supplement: Supplementary file 1 — Additional file 1. [file 12874_2020_1074_MOESM1_ESM.docx]

## **Appendix A**


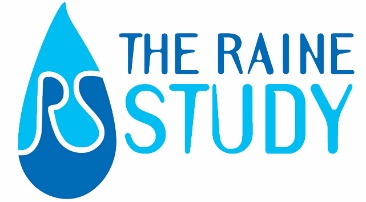
**Semi-structured Interview Guide (Generation 2):**

BENEFITS and BARRIERS

- 1. What has it been like being part of the Raine Study?
- What do you like about being part of the Raine Study?
- What does it feel like to be part of pregnancy cohort study like this?
- Are there things you don’t like about the Raine Study?
  1. Can you recall what it was like when you were younger?
- Can you think of any particularly memorable moments as a participant?
- Did anything ever bother you about participating then?
  1. What do you think kept you coming back…now that you are older?
- Are there things which make it easy/convenient to be involved with the Raine Study? (enabler)
- or difficult to be involved with the Raine Study? (barrier)
- Has anything ever gone really wrong when you’ve attended follow-ups? (any procedures, tests, questionnaires etc..
- Is there anything that has particularly worried or concerned you about the Raine Study? (made you feel uncomfortable?)
- Do you see yourself taking part for much longer? How long might you continue for?

MOTIVATORS

- 1. What could someone SAY or DO (SHOW or GIVE to you) to keep you coming to follow-ups?
- What kind of incentives would you like?
- Do you have any other suggestions for improving your involvement in the Raine Study? During follow-ups or in between time?
- What do you think are good ways to engage children, teens and young adults in studies like Raine?

COMPETITION (POTENTIALLY MERGE WITH BARRIERS)

- 1. What do you think makes people drop out of studies like Raine?
     - What could you say to them if you had a chance to convince them to stay?
     - Have you ever been close to withdrawing?
     - If so, what brought you ‘back’ to the study?
     - How would you feel if you had to withdraw from the study, or if, for some reason, the study came to an end in 2 years?

INFLUENCERS

- 1. Do you ever meet other participants? What’s it like when you do?
- Do you ever talk about the Raine study with other friends or family?
- What do they think about your involvement in the study?
- Tell me about the staff at Raine. How do you get along with them?
- Do you feel like you are a part of something? (if not prompted elsewhere)?

SUMMARY

- 1. If you were asked to promote participation in a new study (like Raine) what would you say to potential participants?
  2. If you could some up your experience as a Raine participant so far, what might you say?
  3. Is there anything that has popped into your mind as we were talking about your experiences that we didn’t get to talk about?
  4. Being part of Raine is like…. (as a summation statement, if not prompted elsewhere)
